# Exploring the Synthetic Chemistry of Phenyl-3-(5-aryl-2-furyl)- 2-propen-1-ones as Urease Inhibitors: Mechanistic Approach through Urease Inhibition, Molecular Docking and Structure–Activity Relationship

**DOI:** 10.3390/biomedicines11092428

**Published:** 2023-08-30

**Authors:** Miraj Fatima, Samina Aslam, Ansa Madeeha Zafar, Ali Irfan, Misbahul Ain Khan, Muhammad Ashraf, Shah Faisal, Sobia Noreen, Gamal A. Shazly, Bakht Ramin Shah, Yousef A. Bin Jardan

**Affiliations:** 1 Department of Chemistry, The Women University, Multan 66000, Pakistan; 2 Department of Chemistry, The Islamia University of Bahawalpur, Bahawalpur 63100, Pakistan; 3 Department of Chemistry, Government Sadiq Women University, Bahawalpur 63100, Pakistan; 4 Department of Chemistry, Government College University Faisalabad, Faisalabad 38000, Pakistan; raialiirfan@gmail.com; 5 Department of Biotechnology and Biochemistry, The Islamia University of Bahawalpur, Bahawalpur 63100, Pakistan; 6 Department of Chemistry, Islamia College University Peshawar, Peshawar 25120, Pakistan; 7 Institute of Chemistry, University of Sargodha, Sargodha 40100, Pakistan; 8 Department of Pharmaceutics, College of Pharmacy, King Saud University, Riyadh 11451, Saudi Arabia; 9 Skin Barrier Research Group, Faculty of Pharmacy in Hradec Králové, Charles University, 500 05 Hradec Králové, Czech Republic

**Keywords:** furan carbaldehyde, Claisen–Schmidt condensation, furan chalcones, urease inhibition, molecular docking, SAR

## Abstract

Furan chalcone scaffolds belong to the most privileged and promising oxygen-containing heterocyclic class of compounds, which have a wide spectrum of therapeutic applications in the field of pharmaceutics, pharmacology, and medicinal chemistry. This research described the synthesis of a series of twelve novel and seven reported furan chalcone (conventional synthetic approach) analogues **4a**–**s** through the application of microwave-assisted synthetic methodology and evaluated for therapeutic inhibition potential against bacterial urease enzyme. In the first step, a series of nineteen substituted 5-aryl-2-furan-2-carbaldehyde derivatives **3a**–**s** were achieved in moderate to good yields (40–70%). These substituted 5-aryl-2-furan-2-carbaldehyde derivatives **3a**–**s** were condensed with acetophenone via Claisen–Schmidt condensation to furnish **19** substituted furan chalcone scaffolds **4a**–**s** in excellent yields (85–92%) in microwave-assisted synthetic approach, while in conventional methodology, these furan chalcone **4a**–**s** were furnished in good yield (65–90%). Furan chalcone structural motifs **4a**–**s** were characterized through elemental analysis and spectroscopic techniques. These nineteen (19)-afforded furan chalcones **4a**–**s** were screened for urease inhibitory chemotherapeutic efficacy and most of the furan chalcones displayed promising urease inhibition activity. The most active urease inhibitors were 1-phenyl-3-[5-(2′,5′-dichlorophenyl)-2-furyl]-2–propen-1-one **4h** with an IC_50_ value of 16.13 ± 2.45 μM, and 1-phenyl- 3-[5-(2′-chlorophenyl)-2-furyl] -2-propen-1-one **4s** with an IC_50_ value of 18.75 ± 0.85 μM in comparison with reference drug thiourea (IC_50_ = 21.25 ± 0.15 μM). These furan chalcone derivatives **4h** and **4s** are more efficient urease inhibitors than reference drug thiourea. Structure–activity relationship (SAR) revealed that the 2,5-dichloro **4h** and 2-chloro **4s** moiety containing furan chalcone derivatives may be considered as potential lead reagents for urease inhibition. The in silico molecular docking study results are in agreement with the experimental biological findings. The results of this study may be helpful in the future drug discovery and designing of novel efficient urease inhibitory agents from this biologically active class of furan chalcones.

## 1. Introduction

**Figure 1:**
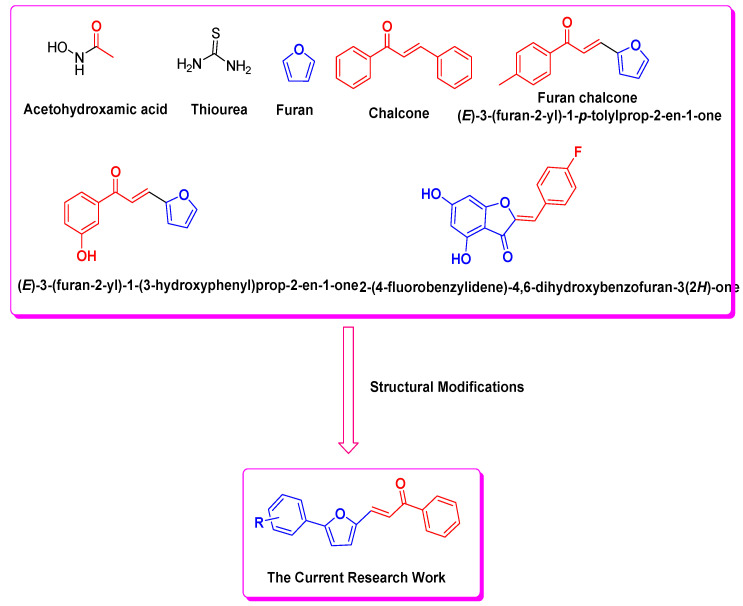
Rationale design of furan chalcones as urease inhibitors [25,26,27,28].

The urease (urea amidohydrolase, EC 3.5.1.5) is a nickel-containing enzyme that catalyzes the hydrolysis of urea to ammonia and carbamate in the final step of nitrogen metabolism [1,2,3]. The carbamate, which is thus formed, rapidly decomposes to yield a second molecule of ammonia. Urease is present in a variety of bacteria (pathogenic as well as soil bacteria), plants, algae, and fungi; therefore, urease is not a native human enzyme. In humans, urease comes from pathogenic bacterial strains such as *Ureaplasma urealyticum*, *Proteus mirabilis*, *Klebsiella pneumoniae*, bacteria from the *Salmonella* and *Staphylococcus* genera, etc., which are responsible for bacterial virulence. These bacterial strains are responsible for many diseases such as urinary tract infections, the formation of urinary stones, peptic ulcers, etc. [4,5]. Ureases are also involved in the development of struvite stone disease, urolithiasis, pyelonephritis, hepatic encephalopathy, hepatic coma, and urinary catheter encrustation [6]. Particularly notorious are the gastric ulcers due to the bacteria *Helicobacter pylori*, whereby urease activity results in an increased pH around the bacteria, aiding in its colonization and protecting it from the other harmful acidic environment of the stomach. Hence, urease inhibitors are effective antibacterial agents, and aid in enhancing the antibacterial effect of other antibiotics. Acetohydroxamic acid (Lithostat, Figure 1) is a well-known example of a clinically used urease inhibitor drug for the treatment of urinary tract infections and certain types of kidney stones.

Generally, natural and synthetic heterocycles are privileged derivatives [7,8], such as furans [9], quinoxalines [10], coumarins [11], benzimidazoles [12], heterocyclic sulfonamides [13], theophyllines [14], imidazoles [15], pyrazoles [16], thiadiazoles [17], etc., which demonstrate wide-spectrum biological activities in the fields of pharmacology, medicinal chemistry, pharmaceutical chemistry, and pharmaceutics. In the same way, various heterocyclic synthetic compounds have been designed to compete with the growing challenges related to ureolytic microorganisms, including thioureas [18], triazoles [19], thiadiazoles [20], benzimidazoles [21], hydroxamic acid [22], phosphoramidate, and thiazolacetamide [23].

Chalcones (Figure 1) are α- and β-unsaturated ketones in which two aromatic rings are joined by three carbon chains, which have a broad spectrum of biological activities such as cytotoxicity, antimitotic, anti-mutagenic, antitumor-promoting activities, antibacterial, antiviral, anti-inflammatory, enzyme inhibition, etc. [24,25,26]. Furan chalcones such as (*E*)-3-(furan-2-yl)-1-*p*-tolylprop-2-en-1-one, (*E*)-3-(furan-2-yl)-1-(3-hydroxyphenyl)pro-2-en-1-one, and aurones (2-(4-fluorobenzylidene)-4,6-dihydroxybenzofuran-3(2*H*)-one) displayed excellent urease inhibition therapeutic efficacy as compared to the reference drug thiourea (Figure 1) [27,28].

In previously published studies, researchers explored the furan derivatives’ chemotherapeutic potential as bacterial tyrosinase inhibitors, human tyrosinase inhibitors, *M. tuberculosis* polyketide synthase 13 inhibitors, anti-oxidants, and anticancer agents [9,24,29,30,31,32,33]. In the present work, structural modifications were carried out to design the synthesis of novel furan chalcone derivatives due to the attractive and promising medicinal and pharmacological profiles of furan and chalcone moieties as cited in the literature [24,25,26,27,28,29,30,31]. The nineteen (19) furan chalcones were synthesized via Claisen–Schmidt condensation reaction by utilizing conventional synthetic approach [31] as well as microwave irradiation synthetic approach. These chalcones were screened against the urease enzyme to discover the inhibitory potential of these 19 furan chalcone derivatives.

## 2. Results and Discussion

### 2.1. Synthetic Chemistry of Furan-Based Chalcones **4a**–**s**

**Scheme 1:**
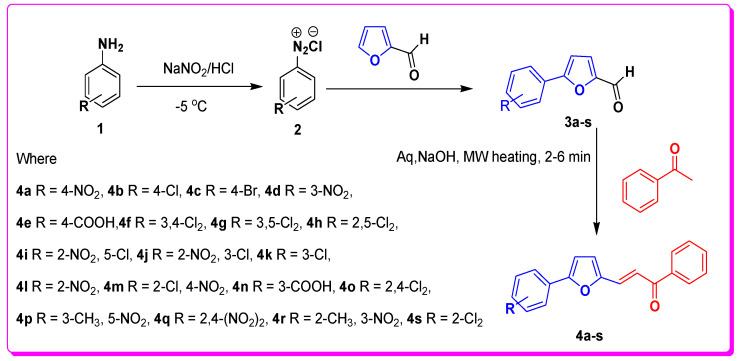
Synthesis of furan moiety-based chalcone derivatives **4a**–**s**.

A series of arylfuran-2-carbaldehydes (**3a**–**s**) and their respective furan chalcone derivatives (**4a**–**s**) were furnished according to the prescribed synthetic approach as depicted in Scheme 1. The arylfuran-2-carbaldehydes **3a**–**s** were afforded in moderate to good yield (40–70%) through a catalytic Meerwein arylation of furfural with arenediazonium salts 2. The best yields in the arylation of furfural were obtained with diazonium salts containing a nitro group or two halogen atoms in the aromatic ring. The Claisen–Schmidt condensation reaction between arylfuran-2-carbaldehydes (**3a**–**s**) and acetophenone under basis (NaOH) catalytic conditions at ambient temperature was carried out in the presence of conventional synthetic approaches [31] as well as microwave irradiation synthetic methodology [33]. The different furan chalcones **4a**–**s** were furnished in excellent yield (85–92%) and good yield (65–90%) by utilizing the microwave-assisted synthetic approach and conventional methodology, respectively. The structures of 19 furan chalcone molecules (**4a**–**s**), percentage yields of both synthetic methodologies, and melting points are displayed in Table 1.

### 2.2. Urease Inhibition Activity of Furan Chalcones **4a**–**s**

Chalcones are important medicinal compounds to combat several pathological conditions associated with ureolytic enzymes (urease). In the literature, it has been reported that the high electron withdrawing effect of the nitro group and the moderate effect of the chloro group could possibly increase the activity of urease inhibitors [35,36]. Considering these facts, we prepared a series of furan chalcones, tested them for their urease inhibitory activities, and found promising urease inhibitory results. Urease inhibition assay was performed according to the reported protocol by Pervez et al. [37].

The furan chalcones **4a**, **4i,** and **4l** displayed IC_50_ values in the range of 90.81 ± 8.99 μM to 91.89 ± 2.24 μM, indicating that these compounds were the least effective against urease enzymes. The furan chalcone scaffolds **4b**, **4c**, **4d**, **4g**, and **4r** were inactive and did not inhibit the urease enzyme as depicted in Table 2. The better urease inhibitory activities (IC_50_ values in the range of 23.09 ± 3.65 μM to 33.96 ± 9.61 μM) were shown by the furan chalone derivatives **4e**, **4j**, **4k**, **4m**, and **4o,** as displayed in Table 2. Furan chalcone derivatives 4n, 4p, and 4q exhibited moderate urease inhibition activity (IC_50_ values in the range of 44.43 ± 6.91 μM to 52.64 ± 8.52 μM) as shown in Table 2. The most active urease inhibitors in our series included 2,5-dichloro functionality, containing 1-phenyl-3-[5-(2′,5′-dichlorophenyl)-2-furyl]-2-propen-1-one **4h,** which has an IC_50_ value of 16.13 ± 2.45 μM, and a 2-chloro moiety-based 1-phenyl-3-[5-(2′-chlorophenyl)-2-furyl]-2-propen-1-one **4s** with an IC_50_ value of 18.75 ± 0.85 μM. The 3,4-dichloro moiety containing 1-phenyl-3-[5-(3′,4′-dichlorophenyl) -2-furyl]-2- propen-1-one **4f** displayed good urease inhibition activity with an IC_50_ value of 21.05 ± 3.2 μM, as compared with the reference standard drug thiourea (Table 2).

### 2.3. Structure–Activity Relationship (SAR) Studies of Furan Chalcones **4a**–**s**

Structural modifications were incorporated by substituting electron-donating and electron-withdrawing functional groups in aromatic rings of furan chalcones **4a–s,** and their influences on the urease inhibitory potential were explored to develop structure–activity relationship (SAR).

**Figure 2:**
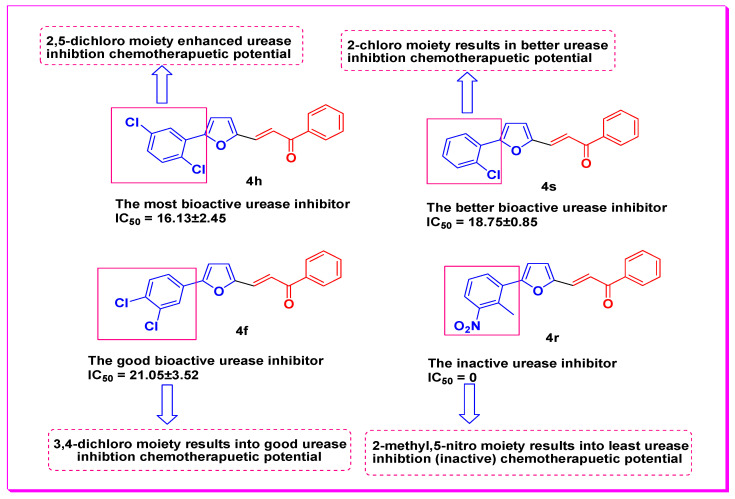
SAR of furan chalcones as urease inhibitors.

In general, furan chalcone compounds containing -Cl and -COOH groups were found to be more active as compared to compounds containing -NO_2_ and -Br groups. Furan chalcone compound **4h** (Figure 2) was the most active inhibitor, having an IC_50_ value of 16.13 ± 2.45 µM. Compounds **4a, 4d,** and **4l** are almost all in which the -NO_2_ group is present at the *ortho*, *meta*, and *para* positions of the phenyl ring. However, for compounds in which the -Cl group is present along with the -NO_2_ group, the activity was found to be enhanced, as in compounds **4j** and **4m** (IC_50_ = 26.05 ± 2.25 µM and 26.71 ± 0.65 µM, respectively). In the same way, when the -Cl group is present at the *para* position of the phenyl ring, as in compound **4b**, this decreases the urease inhibition therapeutic efficacy and the compound becomes inactive, but in compounds **4k** and **4s** (Figure 2), the urease inhibitory activity is enhanced (IC_50_ = 23.09 ± 3.65 µM and 18.75 ± 0.85 µM, respectively) where the -Cl group is present at the *ortho* and *meta* positions of the phenyl ring. Compound **4c** is inactive because the -Br group is present at the *para* position, but when it is replaced by the -COOH group, it shows promising urease inhibition as in compound **4e.** Furan chalcone compound **4h** is the most active inhibitor and has a 2,5-dichloro substituent at the phenyl ring (Figure 2), but if one chloro group is replaced by a -NO_2_ group as in compound **4i,** its activity decreases (IC_50_ value increases to 90.81 ± 8.99 M). Furan chalcone molecule **4g** is urease inactive, in which both chloro groups are *meta* to each other, while its other analogues, 3,4-dichloro containing compound **4f**, 2,5-dichloro containing compound **4h**, and 2,4-dichloro containing compound **4o**, showed promising urease inhibition with IC_50_ values of 21.05 ± 3.52 µM, 16.13 ± 2.45 µM, and 33.96 ± 9.61 µM, respectively. Compound **4e** is inactive because a -NO_2_ group is present at the meta-position of the phenyl group, but compound **4p**, having a 2-methyl-5-nitro substituent on the phenyl group, exhibits an IC_50_ value of 52.64 ± 8.52 µM, whereas its analogue **4r** (Figure 2), having a 2-methyl-3-nitro substituent at the phenyl group, becomes inactive. Furan chalcones having one -NO_2_ group exhibit an IC_50_ of 91.43 ± 6.25 µM as in **4l**; an introduction of a second nitro group on the same phenyl ring has the same effect on the IC_50_ value as in compound **4q**, which has 44.43 ± 6.91 µM, while the furan chalcones having chloro and nitro groups at the *ortho* position show moderate activity.

### 2.4. Molecular Docking, ADMET, and Drug-Likeness Investigations **4a**–**s**

To investigate the probable binding modes and mechanisms of these synthesized compounds, along with the molecular interactions by which these compounds interact with the urease enzyme active site, computational screening studies were performed utilizing the Autodock-Vina (v1.1.2) software. Molecular docking investigations of the top performing compounds in the in vitro studies **4h** and **4s** showed that both of these compounds bind with significantly strong affinities of −7.2 Kcal/mol (Table 3) with the urease enzyme compared to the control thiourea, which showed a binding affinity of −3.4 Kcal/mol (Table 3). The binding conformation and molecular contacts analysis of the compound **4h** showed that the -2′,5′-dichloro phenyl moiety attached to the furan ring binds deep inside the urease active site and is oriented directly at the catalytic dinickel metal active center of this enzyme. Furthermore, the molecular contacts analysis of the compound **4h** revealed that this compound exhibits multiple and diverse types of stronger interactions with the urease enzyme active site.

The molecular interaction analysis of the compound **4h** showed that the -2,5-dichlorophenyl moiety of this compound plays an important role in the robust binding of this compound with the urease enzyme. The analysis showed that the chlorine atoms of the -phenyl ring present at the 2′ and 5′ positions exhibit stronger interactions with important active-site residues of the urease enzyme. One of the chlorine atoms was able to engage with the important catalytic nickel ions via metal-acceptor bonding, along with engaging the HIS138, HIS248, and HIS274 amino acid residues through the alkyl hydrophobic type interaction. Similarly, the second chlorine atom of the -phenyl ring also made an important conventional hydrogen bond interaction with the CYS321 residue, along with an alkyl hydrophobic interaction with the ALA19 urease active site amino acid. Moreover, the -phenyl ring of the compound **4h** also made multiple interactions by engaging the ALA169 and ALA365 via Pi–alkyl-type interactions and the ARG338 via Pi–cation interactions.

**Figure 3:**
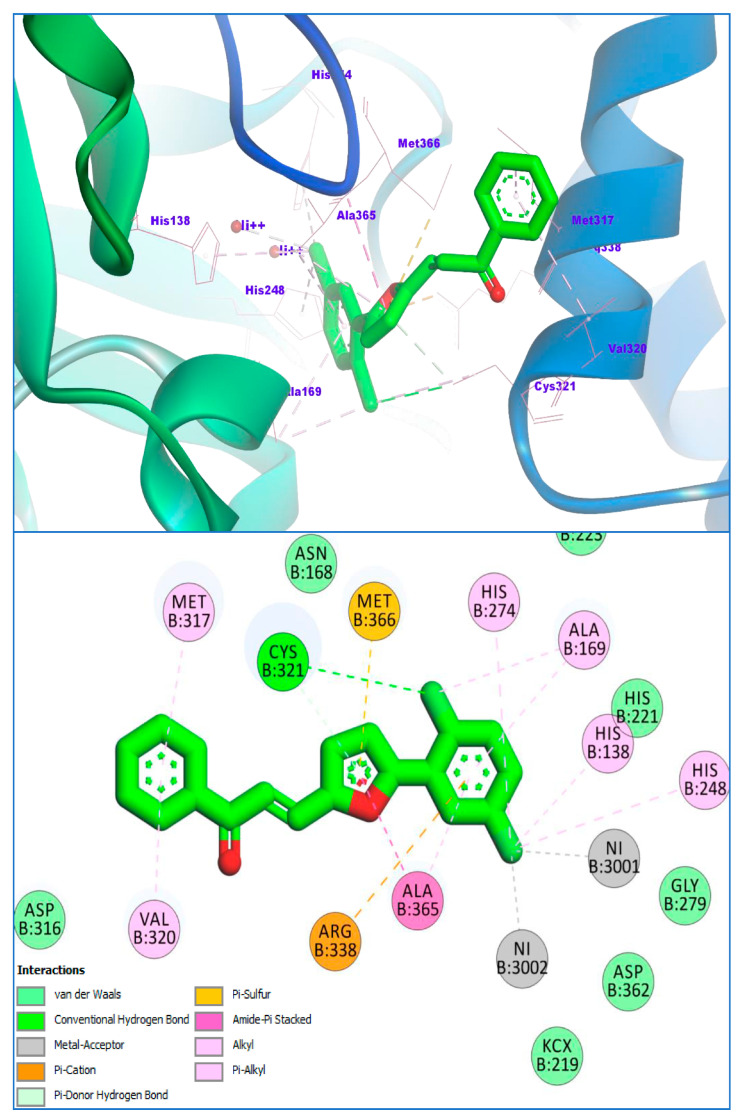
Compound **4h** interacting with urease enzyme active site, upper panel (3D conformation) and lower panel (2D conformation).

Furthermore, the -furan moiety of **4h** also showed multiple interactions with important active-site residues of the urease enzyme. This moiety made a Pi–donor hydrogen bond with the CYS321 amino acid residue, along with a Pi–sulfur-type molecular contact with MET366 and an amide–Pi-stacked interaction with the ALA365 amino acid of the urease active site. The other -phenyl moiety of this compound also contributed two interactions of the Pi–alkyl hydrophobic type by engaging the MET317 and VAL320 outer pocket residues of the urease enzyme active site. Figure 3 contains the two-dimensional and three-dimensional conformational orientations and interaction summary of compound **4h** inside the urease enzyme active site, while Table 3 contains the binding affinities and structures of the investigated compounds.

Previously in the literature, it has been reported that urease inhibitors hamper or inhibit the activity of this enzyme by utilizing different mechanisms [38]. All types of urease enzymes contain an important cysteine amino acid residue. This cysteine residue, which is a part of the flap and is situated on a protein loop that faces the flap at the entry to the active site cavity, is particularly crucial to the way the urease enzyme functions [39,40]. Several inhibitors have been reported that inhibit the urease enzyme by targeting this specific cysteine residue; similarly, several other inhibitors have been reported to directly inhibit this enzyme by targeting and modifying the two nickel ions, which result in a complete inhibition of this enzyme [38].

Keeping in view these previously reported inhibitors and their mode of action and the robust bindings and interactions of the compound **4h,** which was able to interact directly with the important catalytic amino acids, i.e., the flap cysteine and other important histidine residues, and the direct molecular contacts of **4h** with the urease enzyme catalytic nickel ions, it can be inferred from these studies that compound **4h** is a promising candidate for urease inhibition.

#### ADMET Studies

Moreover, ADME and toxicity investigations of compounds **4h** and **4s** were also performed to evaluate their pharmacokinetics and other drug-likeness-related properties. These investigations revealed that these compounds possess favorable gastrointestinal tract absorption if taken orally. These compounds were non-inhibitors of the CYP3A4 and CYP2D6 metabolic enzymes and showed good lipophilicity scores (Log *P*_o/w_ (iLOGP)) along with favorable Log S (ESOL) water solubility scores. These compounds belong to the moderately soluble class of compounds. These compounds also exhibit satisfactory TPSA scores along with good bioavailability values; they also completely followed the Lipinski and Veber drug rules. In the toxicity studies, it was found that these compounds are non-inhibitors of the hERG channel and show no human hepatotoxicity (H-HT); they also show lower probabilities of being AMES and respiratory toxic; moreover, these compounds are also compliant with the Acute Toxicity Rule (ATR) and show zero alerts of toxicity. Table 4 contains some of the discussed ADMET properties and their parameters/values.

## 3. Materials and Methods

All reagents and solvents were obtained from the supplier or recrystallized or redistilled as necessary. Thin-layer chromatography was performed using aluminum sheets (Merck) coated with silica gel 60 F254. Urease enzyme was purchased from Canavalia ensiformis (Jack bean), CAS Number: 9002-13-5. IR spectra were recorded using an IR PerkinElmer Spectrum 1 FTIR spectrophotometer, and peaks were reported as ax (neat)/cm^−1^, which refer to the minimum wave numbers. Proton magnetic resonance spectra were recorded in CDCl_3_ with a Bruker AM 300 spectrometer (Rheinstetten-Forchheim, Germany) operating at 300 MHz, respectively. The ^13^C NMR spectra were recorded in CDCl_3_ with a Bruker AM 100 spectrometer operating at 100 MHz. Tetramethylsilane was used as an internal standard. Elemental analyses for C, H, and N were recorded with the PerkinElmer 2400 Series II CHN Analyzer. Melting points were recorded on a GallenKamp apparatus and are uncorrected.

### 3.1. Synthesis of Furan Chalcone Molecules **4a**–**s**

#### 3.1.1. Synthesis of 5-aryl-2-furaldehydes

Substituted aniline (4.5 g) was dissolved in a mixture of conc. hydrochloric acid and 20 mL of water under stirring and cooled in an ice bath at −5 °C. A solution of sodium nitrite (2 g in 10 mL of water) was added portion-wise, keeping the temperature below 7–8 °C. The reaction mixture was left for an hour for the completion of diazotization, then filtered with the help of glass wool (if any turbidity was observed). Then, a solution of furfural was taken (2 mL in 10 mL of acetone and water), and the diazonium solution was added to it drop by drop. A solution of copper chloride (2 g in 10 mL of water) was also added. The reaction mixture was heated to 30 °C (if necessary) and stirred for 4–6 h, then left for 24 h at room temperature. The precipitates obtained were filtered, dried, and recrystallized with ethanol. By using the above method, various 5-arylfuran-2-carbaldehydes were prepared [41,42,43,44,45].

#### 3.1.2. Synthesis of Furan Chalcones **4a**–**s**

Method A: Conventional Synthetic Approach

Equimolar quantities of aryl furan-2-carbaldehyde (0.001 mol) and appropriate acetophenone (0.001 mol) were taken in an ethanol and water mixture (10 mL ethanol + 10 mL water) in the presence of NaOH as a catalyst in an ice bath (−5 °C), and the mixture was stirred for 4 h. The solid products formed were filtered, dried, and recrystallized from ethanol [31].

Method B: Microwave-Assisted Synthetic Approach

Equimolar quantities (0.001 mol) of 2-acetyl heterocyclic derivatives and respective aldehydes (0.001 mol) were mixed and dissolved in a minimum amount (3 mL) of alcohol. To this, an aqueous sodium hydroxide solution (0.003 mol) was slowly added and mixed. The entire reaction mixture was microwave-irradiated for about 2–6 min at 180 watts [33].

By following Method “A” and Method “B”, various chalcone derivatives (**4a**–**s**) were prepared and their analytical data are given in Supplementary Files.

#### 3.1.3. Furan Chalcones **4a**–**s** Urease Inhibition Assay

The enzyme assay is a modified form of the commonly known Berthelot assay. A total volume of 85 µL assay mixture contained 10 µL of phosphate buffer at pH 7.0 in each well of the 96-well plate, to which 10 µL of sample solution and 25 µL of enzyme solution (0.1347 units) were added. The contents were pre-incubated at 37 °C for 5 min. Then, 40 µL of urea stock solution (20 mM) was added to each well, and incubation continued at 37 °C for another 10 min. After the given time, 115 µL phenol hypochlorite reagent was added to each well (freshly prepared by mixing 45 µL phenol reagent with 70 µL of alkali reagent). For color development, incubation was performed at 37 °C for another 10 min. Absorbance was measured at 625 nm using the 96-well plate reader Synergy HT. The percentage enzyme inhibition was calculated using the following formula:

Inhibition (%) = 100 − (Absorbance of test sample/Absorbance of control) × 100

IC_50_ values (concentration at which 50% enzyme catalyzed reaction occurs) of compounds were calculated using EZ-Fit Enzyme Kinetics Software version 5.03 (Perrella Scientific Inc. Amherst, MA, USA) [46].

#### 3.1.4. Molecular Docking Studies

Molecular docking investigations were performed using the Autodock Vina (v1.1.2) software [47]. The structures of the compounds **4a**–**s** were prepared using the ChemDraw Professional (v16.0) software, and then these structures were energy-minimized using the MOE (v2009.10) software’s MMFF94x forcefield and then saved in the (.mol2) format. These structures were then imported into Autodock Vina, where the necessary Gasteiger charges were added, and then they were saved in the PDBQT format for further screening studies. The protein structure of *E. coli* urease used in this investigation was accessed from the RCSB website with PDB ID 1E9Y [48]. Protein preparation and optimization were performed via MGL Tools (v1.5.7), whereby the H_2_O molecules were removed from the protein molecule, and polar hydrogens and Kollman charges were added. The protein’s binding site (active site) was chosen by setting a grid box located at coordinates (X = 127.83, Y = 129.52, Z = 86.18) with XYZ grid dimensions around the active site of 35 angstroms, and then proceeded with the docking using the AutoDock Vina; all the other parameters were set to default. The binding conformations and molecular interaction analysis of the docked compounds with the urease enzyme were performed using the Biovia Discovery Studio (v2017) software.

#### 3.1.5. ADMET Studies

The ADME and drug-likeness studies were conducted using the SwissADME [49] server, while the toxicity investigations were performed using the ADMETlab 2.0 [50] online server.

## 4. Conclusions

In the present study, a series of twelve novel and seven reported chalcone derivatives (**4a**–**s**) were achieved via an improved microwave-assisted synthetic methodology and evaluated for urease inhibitory chemotherapeutic efficacy. In this series, furan-based chalcone compounds **4h** and **4s** exhibited a comparatively higher urease inhibition chemotherapeutic potential, while others showed moderate urease inhibitory activity as compared to reference standard thiourea. The nature of the substituent on the benzene ring of the aryl group controls the urease inhibition. The most active urease inhibitors were 1-phenyl-3-[5-(2′,5′-dichlorophenyl)-2-furyl]-2-propen-1-one **4h,** having an IC_50_ value of 16.13 ± 2.45 μM, and 1-phenyl-3-[5-(2′-chlorophenyl)-2-furyl]-2-propen-1-one **4s,** with an IC_50_ value of 18.75 ± 0.85 μM in comparison with the reference drug thiourea (IC_50_ = 21.25 ± 0.15 μM). The order of urease inhibition is **4h > 4s > 4f > 4k > 4ae > 4m > 4o > 4aq > 4n > 4p > 4i > 4l > 4a**, and it may act as a potential leading molecule in the drug discovery program. Detailed structure–activity relationship (SAR) and molecular docking studies were carried out to identify the most probable binding site interactions that may lead to the design of even more effective urease inhibitors from this biologically active furan chalcone class of compounds.

## Figures and Tables

**Table 1:** Synthetic data of furan chalcone derivatives **4a**–**s**.

Structures of Compounds	Product Yield (%)		M.P (°C)	
	Method A: Conventional Synthetic Approach [31]	Method B: Microwave Irradiation Synthetic Approach [33]		*Lit* M.P (°C)
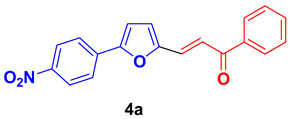	71	85	172–174	172 [31]
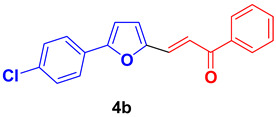	68	90	126–128	126 [31]
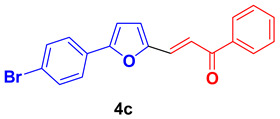	77	90	140–141	140 [31]
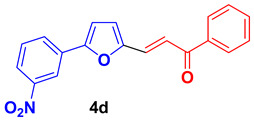	72	88	136–138	136 [31]
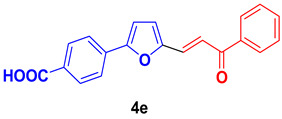	69	85	196–198	200 decompose [31]
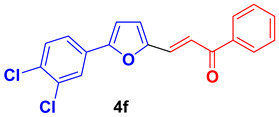	67	90	197–200	-
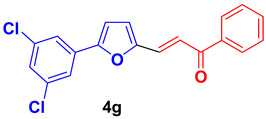	70	88	166–168	-
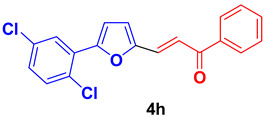	65	82	130–132	-
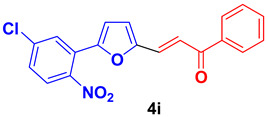	75	90	195–198	-
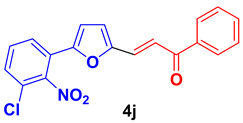	90	92	202–204	-
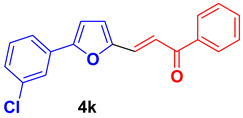	80	90	248–251	-
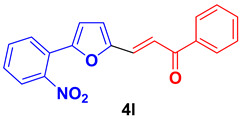	78	90	106–108	106 [34]
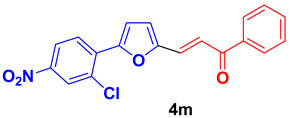	82	88	197–200	-
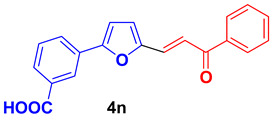	75	80	Decompose ˂ 300	-
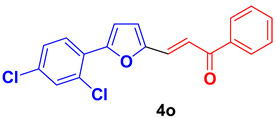	86	z90	116–118	-
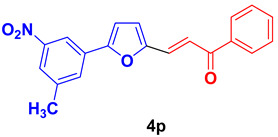	84	92	166–68	-
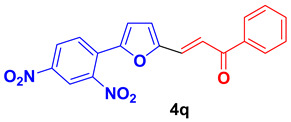	82	90	181–182	-
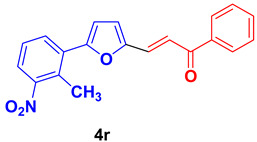	72	86	130–132	-
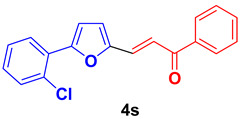	88	90	100–102	96–98 [34]

**Table 2:** Urease inhibition activity of furan chalcone derivatives **4a**–**s**.

Compounds	%Age Urease Inhibition Activity (0.1 mM)	Urease Inhibition IC_50_ (µM)
**4a**	50.71 ± 0.62	91.89 ± 2.24
**4b**	27.15 ± 0.17	-
**4c**	39.41 ± 0.34	-
**4d**	46.55 ± 0.91	-
**4e**	65.46 ± 0.77	24.95 ± 1.77
**4f**	69.71 ± 1.86	21.05 ± 3.52
**4g**	39.72 ± 2.46	-
**4h**	66.24 ± 1.36	16.13 ± 2.45
**4i**	51.72 ± 1.62	90.81 ± 8.99
**4j**	68.24 ± 0.11	26.05 ± 2.25
**4k**	69.52 ± 0.16	23.09 ± 3.65
**4l**	51.47 ± 0.41	91.43 ± 6.25
**4m**	63.14 ± 0.86	26.71 ± 0.65
**4n**	62.28 ± 1.28	46.77 ± 5.44
**4o**	54.88 ± 1.98	33.96 ± 9.61
**4p**	57.37 ± 3.02	52.64 ± 8.52
**4q**	54.74 ± 0.73	44.43 ± 6.91
**4r**	12.64 ± 3.37	-
**4s**	71.41 ± 1.77	18.75 ± 0.85
**Thiourea**		21.25 ± 0.15

**Table 3:** Binding affinities and structures of the investigated compounds against the urease enzyme.

SN	Name	Structure	Binding Affinities with Urease Enzyme
1	**4h**	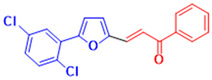	−7.2 Kcal/mol
2	**4s**	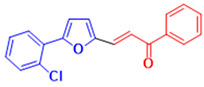	−7.2 Kcal/mol
3	**Thiourea** **(control)**	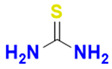	−3.2 Kcal/mol

**Table 4:** ADME and Toxicity profiles along with drug-likeness parameters of compounds **4h** and **4s**.

SN	Compound Name	GI-Tract Absorption	Bioavailability Scores	Lipinski Rule	Water Solubility	Acute Toxicity	Log P_o/w_ (iLOGP)
**1**	**4h**	High	0.55	Accepted	4.87 × 10^−4^ mg/mL	None	3.67
**2**	**4s**	High	0.55	Accepted	1.69 × 10^−3^ mg/mL	None	3.11

## Data Availability

All the data are contained in the manuscript and Supplementary Materials.

## Supplementary Materials

The following supporting information can be downloaded at: https://www.mdpi.com/article/10.3390/biomedicines11092428/s1. NMR and FTIR Spectra are available as supplementary materials.
