# Supplementary material for: Exploring the Synthetic Chemistry of Phenyl-3-(5-aryl-2-furyl)- 2-propen-1-ones as Urease Inhibitors: Mechanistic Approach through Urease Inhibition, Molecular Docking and Structure–Activity Relationship
Source: Biomedicines. 2023 Aug 30;11(9):2428. doi: 10.3390/biomedicines11092428 (PMC10525509; doi:10.3390/biomedicines11092428)
Supplement: Supplementary file 1 [file biomedicines-11-02428-s001.zip › biomedicines-2566281-supplementary.pdf]

Article

Exploring the Synthetic Chemistry of Phenyl-3-(5-aryl-2-furyl)-2-propen-1-ones as Urease Inhibitors: Mechanistic Approach through Urease Inhibition, Molecular Docking and SAR

Miraj Fatima,[1]Samina Aslam[1,2,*,] Ansa Madeeha Zafar[2,3], Ali Irfan[4], Misbahul Ain Khan[2], Muhammad Ashraf[5], Shah Faisal[6], Sobia Noreen[7], Gamal A. Shazly[8], Bakht Ramin Shah[9], Yousef A. Bin Jardan[8,*]

[1]Department of Chemistry, The Women University Multan-2023, Pakistan

[2]Department of Chemistry, The Islamia University of Bahawalpur, Bahawalpur-63100, Pakistan

[3]Department of Chemistry, Government Sadiq Women University, Bahawalpur-63100, Pakistan

[4] Department of Chemistry, Government College University Faisalabad, Faisalabad-38000, Pakistan; raialiirfan@gmail.com (A.I.)

[5] Department of Biotechnology and Biochemistry, The Islamia University of Bahawalpur, Bahawalpur-63100, Pakistan

[6]Department of Chemistry, Islamia College University Peshawar, Peshawar 25120, Pakistan

[7] Institute of Chemistry, University of Sargodha, Sargodha-40100, Pakistan

[8] Department of Pharmaceutics, College of Pharmacy, King Saud University, Riyadh 11451, Saudi Arabia

[9] Skin Barrier Research Group, Faculty of Pharmacy in Hradec Králové,Charles University, Hradec Králové,Czech Republic,

*Correspondence: drsamina.chem@wum.edu.pk (S. A.); ybinjardan@ksu.edu.sa (Y. A. B. J)

Spectral Characterization Data Of Furan Chalcones **4a-4s**

1- 1-phenyl-3-[5-(4'-nitrophenyl)-2-furyl]-2-propen-1-one 4a

Yellow crystalline solid; (FTIR (KBr) (ν, cm^{-1}): 1664.91(C=O conjugated carbonyl group), 15985(C=C conjugated) 1562.44 and 1326.24 (Asym and sym -NO_2), Mass spectrum (EI, 70 eV, I_{rel} %): 319 [M^+] (100) ,273 [M^+- NO_2] (12), 242 [M^+- Ph] (32), 77 [Ph] (23) , 105 [PhCO] (23), 215 [$PhNO_2$furanCH=CH] (55), ^{1}H NMR ($CDCl_3$, 300MHz), δ ppm(*J*, Hz) : 8.53 (1H, d, *J* = 6.3, Ar-H), 7.70 (1H, d, *J* = 6.3, Ar-H), 7.61 (1H, d, *J* = 3.4, Ar-H), 7.57 (1H, d, *J* = 3.4, Ar-H), 7.52(1H, d, J = 4.4, Ar-H), 7.48 (1H, d, *J* = 4.4, Ar-H), 7.40 (1H, d, *J* = 16.3, ethylenic), 7.31 (d1H,d, *J* = 16.3, ethylenic), 6.80 (1H, d, *J* = 2.4, furyl proton), 6.76 (1H, d, *J* = 2.4, furyl proton), ^{13}C NMR ($CDCl_3$, 75 MHz) δ ppm: 189.56 (C=O), 129.90, 128.48 (C=C), 153.58, 152.96, 142.35,141.46, 138.05, 135.36, 132.99, 129.28 128.72, 124.46, 120.59, 118.37,

111.63 (Ar-C) Anal.Calcd.for $C_{19}H_{13}NO_4$: C 71.47; H 4.07; N 4.38, Found C 71.27; H 3.90; N 4.36.

2- 1-phenyl-3-[5-(4'-chlorophenyl)-2-furyl]-2-propen-1-one 4b

Yellow crystalline solid; (FTIR (KBr) (*v*, cm^{-1}1646.14(C=O conjugated carbonyl group), 1585.50 (C=Cconjugated) 1092.68 (C-Cl bond), Mass spectrum(EI,70eV,I_{rel}%): 310[M^++2](27), 308[M^+](80), 273[M^+-Cl](15), 231[M^+-Ph](19), 105[PhCO](18),197[M^+-PhCl](14), ^{1}HNMR($CDCl_3$,300MHz),δppm (*J*,Hz): 8.04(1H,d,*J*=5.7,Ar-H), 7.70(1H,d,*J*=5.7,Ar-H), 7.61(1H,d,*J*=3.0,Ar-H), 7.57(1H,d,*J*=3.0,Ar-H), 7.52(1H,d,*J*=4.2,Ar-H), 7.48(1H,d,*J*=4.2,Ar-H), 7.40(1H,d,*J*=15.0,ethylenic), 7.10(1H,d, *J* =15.0, ethylenic), 6.80(1H, d, *J*= 2.7, furylproton),6.76 (1H,d, *J* =2.7, furyl proton), ^{13}CNMR($CDCl_3$,75MHz)δppm: 189.78(C=O),155.24,151.43(C=C),138.30,134.40,132.74,130.34,129.15,128.63,128.43,125.69,119.12,118.69,108.59(Ar-C) Anal.Calcd.for $C_{19}H_{13}ClO_2$:C 74.01;H 4.22; Found C 73.90;H 4.18.

3- 1-phenyl-3-[5-(4'-bromophenyl)-2-furyl]-2-propen-1-one4c

Yellow crystalline solid; (FTIR (KBr) (v, cm^{-1}): 1666.45 (C=O), 2360.46 (Aromatic ring), 1588.87 (C=Cconjugated) 1029.97 (C-Br bond), Mass spectrum (EI, 70 eV, I_{rel} %): 354 [M^++2] (100), 352 [M^+] (94), 273 [M^+-Br] (28), 197 [M^+-PhBr] (24), 157 [PhBr] (6), 105 [PhCO] (28), ^{1}H NMR ($CDCl_3$,300MHz), δ ppm (J, Hz) : 8.04 (1H, d, J = 5.7, Ar-H), 7.73-7.48 (8H, m, Ar-H), 7.47 (1H, d, J =15.5, ethylenic), 7.24 (1H, d, J = 15.5, ethylenic), 6.79 (1H, d, J = 3.3, furyl proton), 6.77 (1H, d, J = 3.3, furyl proton), ^{13}C NMR ($CDCl_3$, 75 MHz) δ ppm: 189.77 (C=O), 155.23, 151.43 (C=C), 138.24, 132.77, 132.07, 130.34, 128.71, 128.63, 128.43, 125.89, 122.56, 119.09, 118.77,108.70 (Ar-C) Anal.Calcd.for $C_{19}H_{13}BrO_2$: C 64.77; H 3.69; Found C 64.91; H 3.44.

4- 1-phenyl-3-[5-(3'-nitrophenyl)-2-furyl]-2-propen-1-one 4d

Yellow crystalline solid; (FTIR (KBr) (v, cm^{-1}): 1697.58 (C=O conjugated carbonyl group), 1659.96 (C=C conjugated) 1577.72 and 1361.23 (Asym and sym -NO_2), Mass spectrum (EI, 70 eV, I_{rel} %): 319 [M^+] (100), 273 [M^+-NO_2] (6), 197 [M^+-PhNO_2] (31), 105 [PhCO] (20), 77 [Ph] (22), ^{1}H NMR($CDCl_3$, 300MHz), δ ppm (J, Hz) : 8.57 (1H, s, Ar-H), 7.63-7.50 (8H, m, Ar-H), 8.17 (1H, d, J =16.0, ethylenic), 6.93 (1H, d, J = 3.1, furyl proton), 6.84 (1H, d, J = 3.1, furyl proton), 8.05 (1H, d, J = 16.0, ethylenic), ^{13}C NMR ($CDCl_3$, 75 MHz) δ ppm: 189.64(C=O aldehyde), 153.50, 152.30 (C=C), 148.86, 138.06, 132.92, 131.42, 130.00, 129.97, 129.81, 128.70, 128.49, 122.78,120.07, 119.13, 118.37, 110.20 (Ar-C) Anal.Calcd.for $C_{19}H_{13}NO_4$: C 71.47; H 4.07; N 4.38. Found C 71.43; H 4.01; N 4.20.

5- 1-phenyl-3-[5-(4'-phenylcarboxylic acid)-2-furyl]-2-propen-1-one 4e

Yellow crystalline solid; (FTIR (KBr) (v, cm^{-1}): 3347.12 (-OH acid), 1683.25 (C=O acid), 1429.74 (C=C conjugated), 1642.23 (C=O

carbonyl), Mass spectrum (EI, 70 eV, I_{rel} %): 318 [M^+] (1), 105[PhCO] (100), 77 [Ph] (99), 197 [M^+-PhCOOH] (4), 274 [M^+-COOH] (4), ^{1}H NMR($CDCl_3$, 300MHz), δ ppm (J, Hz) : 8.04 (1H, d, J = 5.7, Ar-H), 7.74-7.48 (8H, m, Ar-H), 7.47(1H, d, J = 14.6, ethylenic), 7.13 (1H, d, J = 14.6, ethylenic), 6.80 (1H, d, J = 2.9, furyl proton), 6.76 (1H, d, J = 2.9, furyl proton) ^{13}C NMR ($CDCl_3$, 75 MHz) δ ppm: 189.64 (C=O), 131.89,129.42 (C=C), 168.74 (C=O of acid) 151.72, 151.09, 134.40, 134.14, 130.97, 130.68, 129.87,129.62, 124.88, 123.59, 110.32, 107.59 (Ar-C) Anal.Calcd.for $C_{20}H_{14}O_4$: C 75.47; H 4.40;Found C 75.34; H 4.24.

6- 1-phenyl-3-[5-(3',4'-dichlorophenyl)-2-furyl]-2-propen-1-one 4f

Yellow crystalline solid; (FTIR (KBr) (v, cm^{-1}): 1697.33 (C=O conjugated carbonyl group), 1642.54 (C=C conjugated) 1086.77 (C-Cl bond),Mass spectrum (EI, 70 eV, Irel %): 344 [M^++2] (33), 342 [M^+] (100), 105 [PhCO] (79), 77 [Ph](48), 265 [M^+-Ph] (22) , 197 [M^+-$PhCl_2$] (22), 307 [M^+-Cl] (10) , ^{1}H NMR ($CDCl_3$, 300MHz), δ ppm(J, Hz) : 8.00-7.30 (8H, m, Ar-H), 7.35 (1H, d, J = 15.0, ethylenic), 7.30 (1H,d, J = 15.0, ethylenic), 6.49 (1H, d, J = 3.1, furyl proton), 6.16 (1H, d, J = 3.1, furyl proton), 7.24 1H, s, Ar-H), ^{13}C NMR ($CDCl_3$, 75 MHz) δ ppm : 198.02(C=O aldehyde), 157.20, 150.24 (C=C), 136.72, 133.30, 133.05, 132.77, 130.73, 130.53, 130.47, 128.68, 128.60, 128.15, 125.05,122.60, 108.06, 107.30 (Ar-C) Anal.Calcd.for $C_{19}H_{12}Cl_2O_2$: C 66.66; H 3.50; Found C 66.45;H 3.36.

7- 1-phenyl-3-[5-(3', 5'-dichlorophenyl)-2-furyl]-2-propen-1-one **4g**

Yellow crystalline solid; (FTIR (KBr) (v, cm-1): 1660.64 (C=O conjugated carbonyl group), 1599.51 (C=C conjugated), 1033.99 (C-Cl bond), Mass spectrum (EI, 70 eV, Irel %): 344 [M++2] (12), 342 [M+] (37), 105 [PhCO] (56), 307 [M+-Cl](3), 77 [Ph] (100) , 265 [M+-Ph] (11) , 1H NMR (CDCl3, 300MHz), δ ppm(J, Hz) : 8.06-7.49(5H, m, Ar-H), 7.15 (1H, d, J = 15.7, ethylenic), 7.13 (1H, d, J = 15.7, ethylenic), 7.29 (1H, s,Ar-H), 6.55 (1H, d, J = 3.9, furyl proton), 6.28 1H, d, J = 3.9, furyl proton), 6.80 (2H, d, J = 2.8, Ar-H) , 13C NMR ($CDCl_3$, 75 MHz) δ ppm : 189.68 (C=O aldehyde), 153.25, 152.15 (C=C), 138.10, 135.67, 135.30, 133.26, 133.11, 132.90, 132.48, 130.02, 128.70, 128.16, 122.64, 121.64,119.97, 118.39, 110.08, 108.16 (Ar-C), Anal.Calcd.for C_{19}H12 Cl2O2: C 66.66; H 3.50; Found C 66.61; H 3.28.

8- 1-phenyl-3-[5-(2',5'-dichlorophenyl)-2-furyl]-2-propen-1-one 4h

Yellow crystalline solid; (FTIR (KBr) (v, cm^{-1}): 1696.25 (C=O conjugated carbonyl group), 1644.73 (C=C conjugated) 1088.23 (C-Cl bond), Mass spectrum (EI, 70 eV, I_{rel} %): 344 [M^++2] (2), 342 [M^+] (7), 307 [M^+-Cl] (5), 265 [M^+-Ph] (5),105 [PhCO] (100), 77 [Ph] (39), ^{1}H NMR ($CDCl_3$, 300MHz), δ ppm(J, Hz) : 7.62-7.02 (9H, m,Ar-H),

7.05 (1H, d, J = 16.0, ethylenic), 6.84 (1H, d, J = 2.4, furyl proton), 6.34 (1H, d, J = 2.4, furyl proton), 7.03 (1H, d, J = 16.0, ethylenic), ^{13}C NMR ($CDCl_3$, 75 MHz) δ ppm : 197.18 (C=O aldehyde), 153.35, 151.46 (C=C), 148.63, 138.13, 135.78, 133.20, 133.13, 132.86, 132.10,131.69, 130.09, 129.62, 128.93, 128.69, 128.10, 127.90, 127.90, 127.57 (Ar-C) Anal.Calcd.for$C_{19}H_{12}Cl_2O_2$: C 66.66; H 3.50; Found C 66.57; H 3.34.

9- 1-phenyl-3-[5-(5'-chloro-2'-nitrophenyl)-2-furyl]-2-propen-1-one 4i

Yellow crystalline solid; (FTIR (KBr) (v, cm^{-1}): 1679.26(C=O conjugated carbonyl group), 1642.36 (C=C conjugated) 1466.10 and 1369.26 (Asym and sym $-NO_2$), Mass spectrum (EI, 70 eV, I_{rel} %): 355 [M^++2] (4), 353 [M^+] (11), 307 [M^+-NO_2] (14), 197 [M+-$PhClNO_2$](30), 105[PhCO] (47), 77 [Ph] (32), 131 [PhCOCH=CH] (5), 222 [M^+-131] (11) , ^1H NMR ($CDCl_3$,300MHz), δ ppm(J, Hz) : 7.28-7.24 (7H, m, Ar-H), 7.56 (1H, d, J = 14.4, ethylenic), 6.59 (1H,d, J = 3.3, furyl proton), 6.22 (1H, d, J = 3.3, furyl proton), 7.46 (1H, d, J = 14.4, ethylenic), 7.98 (1H, s, Ar-H) ^{13}C NMR ($CDCl_3$, 75 MHz) δ ppm : 188.34(C=O aldehyde), 131.40, 128.88(C=C), 155.54, 151.78, 139.30, 134.36, 133.20, 132.41, 131.65, 131.11, 130.90, 129.92, 129.48, 120.97,119.69, 115.31, 110.82(Ar-C) Anal.Calcd.for $C_{19}H_{12}ClNO_4$: C 64.58; H 3.39; N 3.96 Found C64.52; H 3.31; N 3.89.

10- 1-phenyl-3-[5-(4'-chloro-2'-nitrophenyl)-2-furyl]-2-propen-1-one 4j

Yellow crystalline solid; (FTIR (KBr) (v, cm^{-1}): 1697.58 (C=O conjugated carbonyl group), 1659.96 (C=C conjugated) 1577.72 and 1361.23 (Asym and sym $-NO_2$), 1109.01 (C-Cl bond), Mass spectrum (EI, 70 eV, I_{rel} %): 355 [M^++2] (2), 353[M^+] (6), 157 [PhClNO2] (16) , 105 [PhCO] (100), 77 [Ph] (40), 222 [M^+-131] (6) ,1H NMR($CDCl_3$, 300MHz), δ ppm(J, Hz) : 8.53-7.38 (8H, m, Ar-H), 7.34 (1H, d, J = 15.8, ethylenic),7.31 (1H, d, J = 15.8, ethylenic), 7.31 (1H, d, J = 2.1, furyl proton), 6.63 (1H, d, J = 2.1, furyl proton) ^{13}C NMR ($CDCl_3$, 75 MHz) δ ppm : 188.78(C=O aldehyde) 131.87, 126.90 (C=C), 156.78, 151.89, 133.27, 128.65, 128.17, 110.78, 107.56 (Ar-C) Anal.Calcd.for $C_{19}H_{12}ClNO_4$: C64.58; H 3.39; N 3.96 Found C 64.24; H 3.01; N 3.68.

11- 1-phenyl-3-[5'-(3''-chlorophenyl)-2-furyl]-2-propen-1-one 4k

Yellow crystalline solid; (FTIR (KBr) (v, cm^{-1}): 1647.00 (C=O conjugated carbonyl group), 1596.38 (C=C conjugated), 1091.05 (C-Cl bond), Mass spectrum (EI, 70 eV, I_{rel} %): 310 [M^++2] (32), 308 [M+] (90), 273 [M+-Cl] (9), 231 [M+-Ph] (17), 105 [PhCO] (100), 178[PhClfuran] (7), 77 [Ph] (71),^1H NMR ($CDCl_3$, 300MHz), δ ppm(J, Hz) : 7.56-7.36 (9H, m, Ar-H), 7.34 (1H, d, J = 15.2, ethylenic), 6.50 (1H, d, J = 15.2, ethylenic), 6.15 (1H, d, J = 3.7, furyl proton), 7.15 (1H, d, J = 3.7, furyl proton), ^{13}C NMR (CDCl3, 75 MHz) δ ppm :

198.09(C=O aldehyde), 156.89, 151.18 (C=C), 136.85, 134.61, 133.22, 133.11, 133.03, 132.55, 129.82, 129.25, 128.66, 128.61, 128.17, 126.85, 121.53, 107.95, 106.92(Ar-C) Anal.Calcd.for $C_{19}H_{13}ClO_2$: C 74.02; H 4.22; Found C 73.72; H 3.98.

12- 1-phenyl-3-[5-(2'-nitrophenyl)-2-furyl]-2-propen-1-one 4l

Yellow crystalline solid; (FTIR (KBr) (v, cm^{-1}): 1694.31 (C=O conjugated carbonyl group), 1610.33(C=C conjugated), 1586.82 and 1367.07 (Asym and sym $-NO_2$), Mass spectrum (EI, 70 eV, I_{rel} %): 319 [M^+] (2), 272 [M^+-NO_2] (10), 105 [PhCO] (100). 77 [Ph] (29),^{1}H NMR ($CDCl_3$, 300MHz), δ ppm(J, Hz) : 7.99-7.42 (9H, m, Ar-H), 7.35 (1H, d, J = 15.4,ethylenic), 6.54 (1H, d, J = 2.1, furyl proton), 6.20 (1H, d, J = 2.1, furyl proton), 7.10 (1H, d, J = 15.4, ethylenic)^{13}C NMR ($CDCl_3$, 75 MHz) δ ppm : 197.99(C=O aldehyde), 158.39, 150.14(C=C), 133.21, 131.61, 128.63, 128.20, 127.77, 123.68, 110.43, 108.10 (Ar-C) Anal.Calcd.for$C_{19}H_{13}NO_4$: C 71.47; H 4.07; N 4.38 Found C 71.61; H 4.28; N 4.45.

13- 1-phenyl-3-[5-(2'-chloro-4'-nitrophenyl)-2-furyl]-2-propen-1-one 4m

Yellow crystalline solid; (FTIR (KBr) (v, cm^{-1}): 1662.92 (C=O conjugated carbonyl group), 1586.65(C=C conjugated), 1467.27 (Asym and sym $-NO_2$), 1100.81 (C-Cl bond), Mass spectrum (EI, 70 eV, I_{rel}%): 355 [M^++2] (15), 353 [M^+] (41), 191 [$PhClNO_2$furan](32), 122 [$PhClNO_2$] (15), 105 [PhCO] (100),^{1}H NMR ($CDCl_3$, 300MHz), δ ppm(J, Hz) : 8.35(1H, s, Ar-H), 8.29 1H, d, J = 5.7, Ar-H), 8.20 (1H, d, J = 15.7, ethylenic), 8.04 (1H, d, J = 6.3,Ar-H), 7.99 (1H, d, J = 6.3, Ar-H), 7.79 (1H, d, J = 7.8, Ar-H), 7.71 (1H, d, J = 7.8, Ar-H), 7.60(1H, d, J = 3.3, Ar-H), 7.54 (1H, d, J = 15.7, ethylenic), 7.15 (1H, d, J = 3.3, Ar-H), 6.89 (1H, d,J = 3.1, furyl proton), 6.30 (1H, d, J = 3.1, furyl proton) ^{13}C NMR ($CDCl_3$, 75 MHz) δ ppm :187.89(C=O aldehyde), 130.24, 127.95 (C=C), 134.54, 131.18, 129.83, 129.62, 129.30, 128.69,128.59, 128.19, 127.03, 124.98, 123.04, 116.69, 109.73 (Ar-C) Anal.Calcd.for $C_{19}H_{12}$ $ClNO_4$: C64.58; H 3.39; N 3.96 Found C 64.35; H 3.45; N 3.58.

14- 1-phenyl-3-[5-(3'-phenylcarboxylic acid)-2-furyl]-2-propen-1-one 4n

Yellow crystalline solid;((KBr) (v, cm^{-1}): 1644.73 (C=O), 1464.64 (C=Cconjugated), 3109.35 (-OH acid), 1696.25 (C=O of acid), Mass spectrum (EI, 70 eV, I_{rel} %): 318 [M^+] (75), 273 [M^+-COOH] (22), 197 [M^+-PhCOOH] (15), 105 [PhCO] (100),^{1}H NMR($CDCl_3$, 300MHz), δ ppm(J, Hz) : 10.12 (1H, s, COOH), 8.35-8.12 (3H, m, Ar-H), 8.09 (1H, s,Ar-H), 7.48-7.29 (4H, m, Ar-H), 7.93 (1H, d, J = 16.2, ethylenic), 7.23 (1H, d, J = 2.9, furylproton), 7.67 (1H, d, J = 16.2, ethylenic), 6.92 (1H, d, J = 2.9, furyl proton), ^{13}C NMR ($CDCl_3$, 75 MHz) δ ppm : 189.54(C=O aldehyde), 165.44 (C=O acid) 131.86,

127.11 (C=C), 155.43,151.69, 137.75, 133.89, 130.23, 129.88, 122.45, 121.78, 119.34, 110.37(Ar-C) Anal.Calcd.for$C_{20}H_{14}$ O^4: C 75.47; H 4.40; Found C 75.67; H 4.58.

15- **1-phenyl-3-[5-(2',4'-dichlorophenyl)-2-furyl]-2-propen-1-**one **4o**

Yellow crystalline solid; (FTIR (KBr) (v, cm-1): 1697.97 (C=O), 1642.20 (C=C conjugated) , 1086.66 (C-Cl bond), Mass spectrum (EI, 70 eV, I_{rel} %): 344 [M^++2] (10), 342 [M^+] (28), 307 [M^+-Cl](10), 197 [M^+-PhCl2] (32), 139 [PhCOCl] (15), 105 [PhCO] (74) ^{1}H NMR ($CDCl_3$, 300MHz), δppm(J, Hz) : 7.50-7.91 (8H, m, Ar-H), 7.47 (1H, d, J = 15.3, ethylenic), 6.79 (1H, d, J = 15.3,ethylenic), 6.80 (2H, d, J = 3.3, furyl proton) ^{13}C NMR ($CDCl_3$, 75 MHz) δ ppm : 188.56(C=O aldehyde), 130.32, 129.77 (C=C), 153.60, 151.08, 137.43, 133.39, 133.21, 128.71, 127.92,127.70, 126.47, 119.90, 110.02 (Ar-C) Anal.Calcd.for $C_{19}H_{12}Cl_2O_2$: C 66.66; H 3.50; Found C66.10; H 3.63.

16- **1-phenyl-3-[5-(2'-methyl-5'-nitrophenyl)-2-furyl]-2-propen-1-**one **4p**

Yellow crystalline solid; (FTIR (KBr) (v, cm^{-1}): 1668.87 (C=O conjugated carbonyl group), 1586.65 (C=C conjugated) 1530.78 and 1351.23 (Asym and sym -NO_2), Mass spectrum (EI, 70 eV, I_{rel}%): 331 [M^+] (34), 318 [M^+-CH_3] (45), 287 [M^+-NO_2] (10), 197 [M^+-136] (18), 105 [PhCO](100),^{1}H NMR ($CDCl_3$, 300MHz), δ ppm(J, Hz) : 8.15-8.03 (4H, m, Ar-H), 8.11 (1H, d, J = 8.7,Ar-H), 7.72 (1H, d, J = 8.7, Ar-H), 7.69 (4H, m, Ar-H), 7.60 (1H, d, J = 15.9, ethylenic), 7.33(1H, d, J = 3.6, furyl proton), 7.23 (1H, d, J = 3.6, furyl proton),7.47 (1H, d, J = 15.9, ethylenic), 2.12 (3H, s, CH_3)^{13}C NMR ($CDCl_3$, 75 MHz) δ ppm : 188.71 (C=O), 130.00, 128.32 (C=C),21.84 (CH3), 152.76, 151.43, 146.28, 142.64, 137.49, 133.10, 132.99, 129.73, 128.67, 122.60,121.40, 119.80, 118.94, 114.08 (Ar-C), 15.43 (CH_3)Anal.Calcd.for $C_{20}H_{15}NO_4$: C 72.07; H 4.50;N 4.20 Found C 71.78; H 3.91; N 4.01.

17- 1-phenyl-3-[5-(2', 4'-dinitrophenyl)-2-furyl]-2-propen-1-one 4q

Yellow crystalline solid; (FTIR (KBr) (v, cm^{-1}): 1659.34cm^{-1}(C=O conjugated carbonyl group), 1591.21 cm^{-1}(C=C conjugated), 1552.82 and 1357.17 cm^{-1} (Asym and sym -NO_2), Mass spectrum (EI, 70 eV, I_{rel} %): 364 [M^+] (100), 318 [M^+-NO_2] (55), 197 [M^+-167] (18), 167 [M^+-Ph (NO_2)$_2$] (10), 105 [PhCO] (45),^{1}H NMR ($CDCl_3$, 300MHz), δ ppm(J, Hz) : 9.10 (1H, s, Ar-H), 6.80 (1H, d, J = 3.1, furyl proton), 7.00 (1H, d, J = 3.1, furyl proton), 7.51-7.70 (5H, m, Ar-H), 8.10 (2H, d, J = 4.3, Ar-H), 8.30 (1H, s, Ar-H), 8.42 (1H, s, Ar-H), 7.34 (1H, d, J = 15.4,ethylenic), 6.90 (1H, d, J = 15.4, ethylenic),^{13}C NMR ($CDCl_3$, 75 MHz) δ ppm : 188.58 (C=O), 152.45, 149.62 (C=C) 146.46, 137.31,

133.31, 132.84, 129.63, 129.53, 129.04, 128.87, 128.46,126.04, 122.56, 121.19, 119.12, 117.18 (Ar-C) Anal.Calcd.for $C_{19}H_{12}N_2O_6$: C 62.63; H 3.29; N7.69 Found C 62.42; H 3.10; N 6.99.

18- **1-phenyl-3-[5-(2'-methyl-3'-nitrophenyl)-2-furyl]-2-propen-1-**one **4r**

Yellow crystalline solid; (FTIR (KBr) (v, cm^{-1}): 1697.58 (C=O), 1659.95(C=C conjugated) 1577.72 and 1361.23 (Asym and sym -NO_2), Mass spectrum (EI, 70 eV, I_{rel}%): 333 [$M^{+]}$ (20), 286 [M^+- NO_2] (5), 77 [Ph] (100), 215 [M^+-PhCOCH] (10), 197 [M^+-$PhCH_3NO_2$] (10), 157 [286- PhCOCHCH] (75), 105 [PhCO] (58),^{1}H NMR ($CDCl_3$, 300MHz), δ ppm(J, Hz) : 8.15-7.68 (9H, m, Ar-H), 7.67 (1H, d, J = 16.7, ethylenic), 7.57 (1H, d, J = 16.7, ethylenic), 7.28 (1H, d, J = 2.9,furyl proton,), 7.11 (1H, d, J = 2.9, furyl proton), 2.52 (3H, s, CH3),^{13}C NMR ($CDCl_3$, 75 MHz) δ ppm : 188.56 (C=O), 131.42, 127.95 (C=C), 16.53 (CH_3), 153.06, 151.99, 151.38, 137.44,133.14, 131.65, 129.98, 128.84, 128.68, 127.34, 119.50, 114.36 (Ar-C),Anal.Calcd.for $C_{20}H_{15}NO_4$: C 72.07; H 4.50; N 4.20 Found C 72.01; H 3.92; N 4.02

19- 1-phenyl-3-[5-(2'-chlorophenyl)-2-furyl]-2-propen-1-one 4s

Yellow crystalline solid; (FTIR (KBr) (v, cm^{-1}): 1694.31 (C=O), 1586.82 (C=C conjugated), 1030.49 (C-Cl bond), Mass spectrum (EI, 70 eV, I_{rel} %): 310 [M^++2] (11), 308 [$M_{+]}$ (38), 273[M^+-Cl] (76), 139 [PhCOCl] (100), 197 [M^+-PhCl] (12), 111 [PhCl] (46), 105 [PhCO] (58)^{1}HNMR ($CDCl_3$, 300MHz), δ ppm(J, Hz) : 7.67-7.27 (9H, m, Ar-H), 7.76 (1H, d, J = 16.7,ethylenic), 7.71 (1H, d, J = 16.7, ethylenic), 7.25 (1H, d, J = 2.9, furyl proton,), 7.20 (1H, d, J = 2.9, furyl proton) ^{13}C NMR ($CDCl_3$, 75 MHz) δ ppm : 187.65 (C=O), 131.44, 127.34 (C=C),155.29, 151.36, 137.55, 133.25, 132.19, 130.78, 129.46, 126.45, 122.74, 118.90, 110.68 (Ar-C),Anal.Calcd.for $C_{19}H_{13}ClO_2$: C 74.02;H 4.22; Found C 73.85; H 3.99.
